# Dynamic organelle localization and cytoskeletal reorganization during preimplantation mouse embryo development revealed by live imaging of genetically encoded fluorescent fusion proteins

**DOI:** 10.1002/dvg.23277

**Published:** 2019-01-13

**Authors:** Hiroshi Kiyonari, Mari Kaneko, Takaya Abe, Go Shioi, Shinichi Aizawa, Yasuhide Furuta, Toshihiko Fujimori

**Affiliations:** ^1^ Laboratory for Animal Resource Development RIKEN Center for Biosystems Dynamics Research Kobe Japan; ^2^ Laboratory for Genetic Engineering RIKEN Center for Biosystems Dynamics Research Kobe Japan; ^3^ Division of Embryology National Institute for Basic Biology (NIBB) Okazaki Japan

**Keywords:** conditional, knock‐in, organelle, reporter mouse, ROSA26, time‐lapse

## Abstract

Live imaging is one of the most powerful technologies for studying the behaviors of cells and molecules in living embryos. Previously, we established a series of reporter mouse lines in which specific organelles are labeled with various fluorescent proteins. In this study, we examined the localizations of fluorescent signals during preimplantation development of these mouse lines, as well as a newly established one, by time‐lapse imaging. Each organelle was specifically marked with fluorescent fusion proteins; fluorescent signals were clearly visible during the whole period of time‐lapse observation, and the expression of the reporters did not affect embryonic development. We found that some organelles dramatically change their sub‐cellular distributions during preimplantation stages. In addition, by crossing mouse lines carrying reporters of two distinct colors, we could simultaneously visualize two types of organelles. These results confirm that our reporter mouse lines can be valuable genetic tools for live imaging of embryonic development.

## 1 INTRODUCTION

Recent progress in live imaging technology has made it possible to visualize not only phenomena in vivo but also the precise behaviors of biological molecules in living cells. The development of image acquisition systems, including microscopes and detectors, along with improved image analysis technologies, has attracted a great deal of attention in basic life science, as well as in biotechnology and biomedical engineering. In parallel with the development of these imaging technologies, various fluorescent proteins have been developed (Bevis & Glick, 2002; Bulina et al., 2006; Chalfie, Tu, Euskirchen, Ward, & Prasher, 1994; Chudakov, Matz, Lukyanov, & Lukyanov, 2010; Hoshino, Nakajima, & Ohmiya, 2007; Shaner et al., 2004; Shimomura, Johnson, & Saiga, 1962; Zhang, Gurtu, & Kain, 1996) which can readily be applied to transgenic techniques in a wide variety of organisms. This combination of technologies allows us to observe living cells continuously and in detail, enabling closer analysis in diverse biological phenomena. This has also made it possible to examine the behaviors of cells and molecules involved in cell division, cell movement, cell death, morphological changes of cellular sheets in embryos, gene expression, and localization of various drugs and chemical substances in living animals.

Although a diverse array of reporter mice expressing fluorescent proteins are available (Abe & Fujimori, 2013), many of them are often difficult to use due to inherent problems in unstable reporter expression associated with variable transgene copy numbers, positional effects, or silencing over the course of multiple generations (Trichas, Begbie, & Srinivas, 2008). To overcome these problems, we previously established a series of conditional reporter mouse lines by homologous recombination in ES cells at the ROSA26 locus (Abe et al., 2011; Katsunuma et al., 2016; Shioi et al., 2011), warranting a single‐copy reporter transgene in the defined gene locus. Following excision of the stop sequence by the Cre recombinase, specific organelles can be visualized by monitoring green or red fluorescent proteins. In this study, we observed the behaviors of fluorescent reporter proteins during preimplantation development by live imaging of embryos obtained from these reporter mouse lines, as well as a novel Lyn‐mCherry fusion protein reporter line in which the plasma membrane can be visualized.

## 2 RESULTS

### 2.1 Time‐lapse observation of preimplantation development of R26 reporter mice

**Figure 1:**
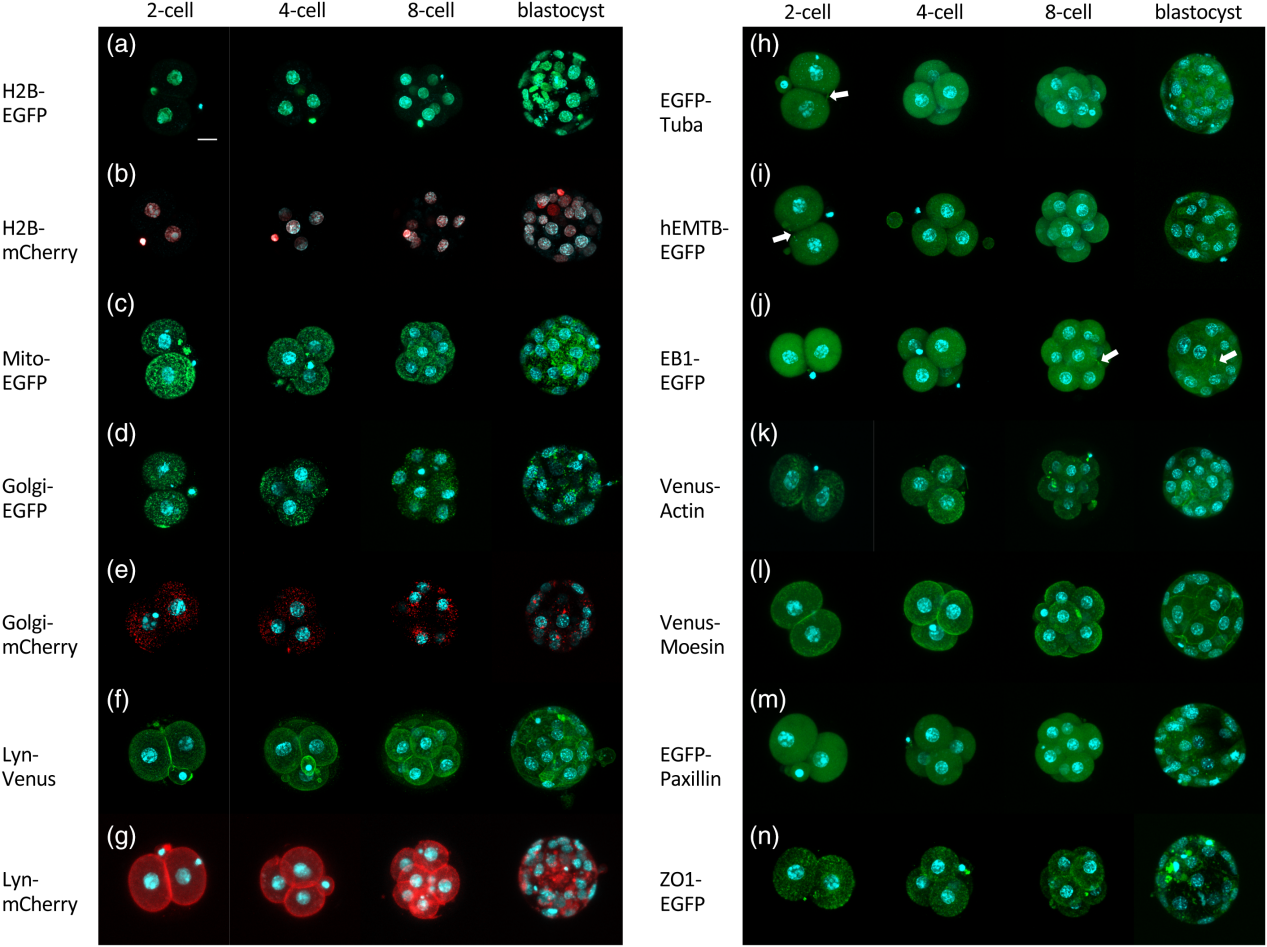
Expression of fluorescent fusion proteins from the two2‐cell through blastocyst stages in a series of reporter mice. (a) H2B‐EGFP, (b) H2B‐mCherry, (c) Mito‐EGFP, (d) Golgi‐EGFP, (e) Golgi‐mCherry, (f) Lyn‐Venus, (g) Lyn‐mCherry, (h) EGFP‐Tuba, (i) hEMTB‐EGFP, (j) EB1‐EGFP, (k) Venus‐Actin, (l) Venus‐Moesin, (m) EGFP‐Paxillin, and (n) ZO1‐EGFP. All images are three‐dimensional reconstruction images. All nuclei were stained with Hoechst 33342. White arrows in (h, i, and j) indicate midbody microtubule. The z‐step size is 2.5 μm. Scale bar = 20 μm

**Figure 2:**
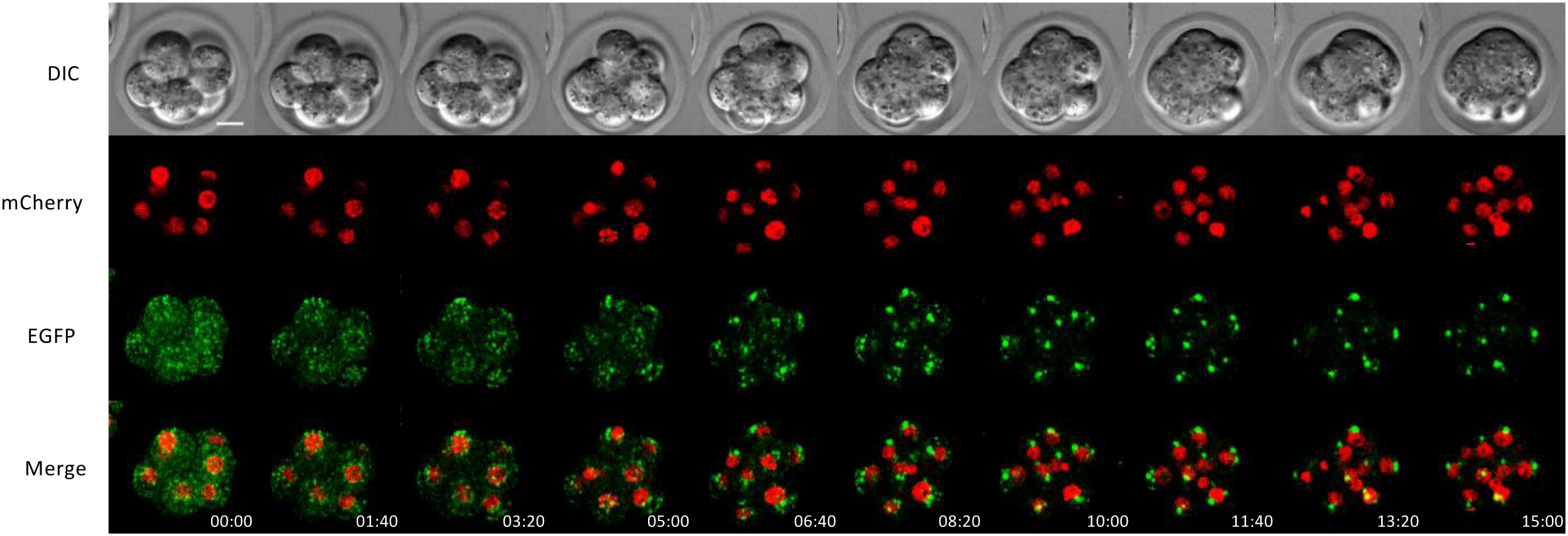
Time‐lapse images of an eight‐cell R26‐Golgi‐EGFP/H2B‐mCherry embryos. The eight‐cell embryos obtained by crossing R26‐Golgi‐EGFP and R26‐H2B‐mCherry mice were subjected to time‐lapse observation. Snapshot images are from time‐lapse observations taken at 2‐min intervals. Golgi‐EGFP, H2B‐mCherry, and merged images are maximum intensity projections (MIPs) compiled from Z‐series images. Selected snapshots taken at 100‐min intervals are shown. Time‐stamps are shown on the bottom. The z‐step size is 3 μm. Scale bar = 20 μm

**Figure 3:**
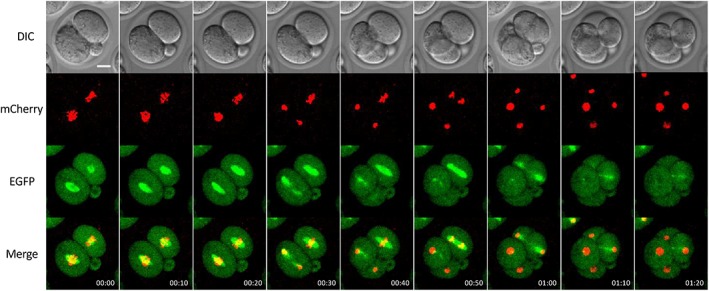
Time‐lapse images of spindles and chromosomes during cell division. Time‐lapse images of cell divisions from the two‐cell and four‐cell stages. The embryo was obtained by crossing R26‐EGFP‐Tuba and R26‐H2B‐mCherry mice. Time‐lapse images were taken every 2 min; selected images at 10‐min intervals (i.e., every five images) are shown. Time‐stamp are shown on the bottom. H2B‐mCherry, EGFP‐Tuba, and merged images are MIPs. The z‐step size is 5 μm. Scale bar = 20 μm

**Figure 4:**
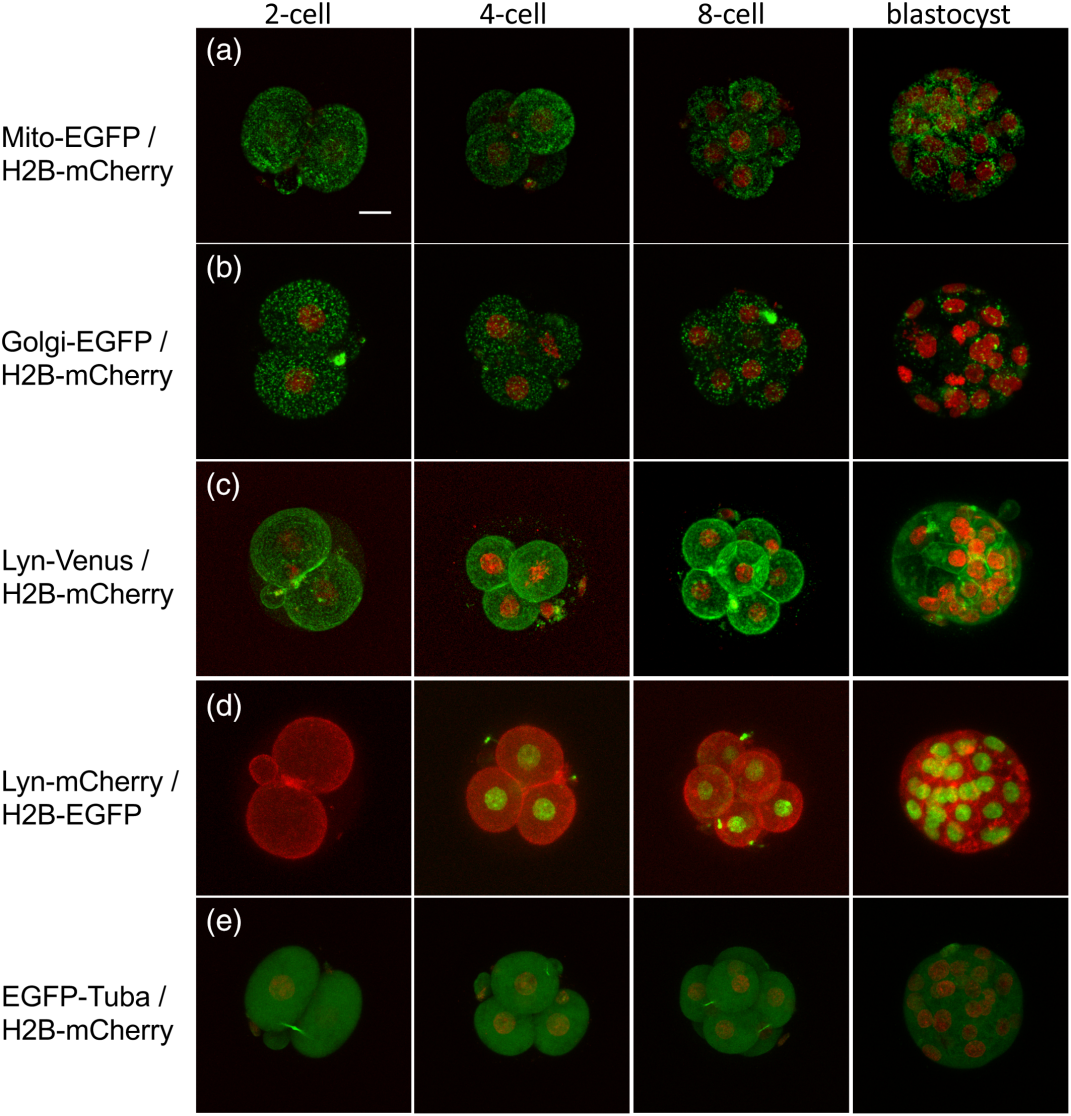
Localization of fluorescent fusion proteins in dual‐labeled embryos. Localization of fluorescent fusion proteins was examined in two‐cell, four‐cell, eight‐cell, and blastocyst stage embryos obtained by crossing between (a) R26‐Mito‐EGFP and R26‐H2B‐mCherry, (b) R26‐Golgi‐EGFP and R26‐H2B‐mCherry, (c) R26‐Lyn‐Venus and R26‐H2B‐mCherry, (d) R26‐Lyn‐mCherry and R26‐H2B‐EGFP, and (e) R26‐EGFP‐Tuba and R26‐H2B‐mCherry mice. All images are MIPs. The z‐step size is 2.5 μm. Scale bar = 20 μm

To observe the behaviors of fluorescent fusion proteins in preimplantation embryos from the two‐cell through blastocyst stages, we used two‐cell embryos from R26 reporter mouse lines that lack the neo cassette; these mice, which were generated by crossing homozygous R26R mice (Abe et al., 2011; Katsunuma et al., 2016; http://www2.clst.riken.jp/arg/reporter_mice.html) with Ella‐Cre transgenic mice (Lakso et al., 1996), express respective fluorescent fusion proteins ubiquitously. These embryos lacked any obvious abnormalities in embryonic development, as previously reported (Abe et al., 2011). To closely observe subcellular structures, we used a conventional laser scanning confocal microscope (Nikon A1‐Ti: Tokyo, Japan) (Figures 1 and 4; Supporting Information Figures S1, S4–S6 and Movies S1, S5). Embryos of all reporter mouse lines were subjected to time‐lapse imaging during the preimplantation stages for up to 72 hr. We cultured embryos and captured time‐lapse images over the course of development using an LCV100 Olympus incubation imaging system equipped with a CSU10 spinning disc confocal system (Yokogawa) and an iXon+EMCCD camera (Andor) (Figures 2 and 3; Supporting Information Figures S2, S3, S7a and Movies S2–S4) or with a CV1000 spinning disc confocal imaging system (Yokogawa) equipped with an EM‐CCD camera (Hamamatsu) (Supporting Information Figure S7c). Embryonic development was not apparently disrupted by continuous time‐lapse observation; because the total cell numbers of blastocyst stage embryos (66 hr after thawing) were not significantly different between time‐lapse imaged Lyn‐Venus embryos (average: 38.6 cells ± 4.3), non‐imaged Lyn‐Venus embryos (average: 37.9 ± 2.9 cells) and wild‐type embryos (average: 34.6 ± 4.6). The timing of cell division was also similar between embryos with and without transgenes (data not shown).

#### 2.1.1 Nucleus

R26‐H2B‐EGFP (Abe et al., 2011; Accession (Acc.) No. CDB0238K) and R26‐H2B‐mCherry (Abe et al., 2011; Acc. No. CDB0239K) were used to label chromosomes and to visualize nuclei by monitoring green and red fluorescence, respectively. Expression of both fluorescent fusion proteins in homozygous embryos was strong enough to enable visualization of all nuclei and yielded a pattern similar to that of Hoechst 33342 (Invitrogen: Thermo Fisher, Waltham, MA, USA) staining confirming these reporters can be used reliably for chromosome labeling (Figure 1a,b; Supporting Information Figure S1a–b′ and Movie S2a,b). Importantly, these fluorescent signals were observed during mitosis, and segregating chromosomes were also clearly labeled.

#### 2.1.2 Mitochondria

In R26‐Mito‐EGFP (Abe et al., 2011; Acc. No. CDB0251K) homozygous embryos, strong EGFP signals manifested as cytoplasmic spots, and these signals localized to regions near the apical plasma membrane in the cytoplasm in 2–8‐cell embryos (Figure 1c; Supporting Information Figure S1c,c′ and Movie S2c). The localization of signals in early preimplantation embryos was similar to the reported localization of mitochondria (Basu et al., 2008; Kawamura et al., 2005; Lee, Hwang, Sun, Xu, & Kim, 2011). In blastocysts, EGFP signals mainly localized at the periphery of nuclei (Figure 1c; Supporting Information Movie S1a). In addition, the Mito‐EGFP signals in this mouse line were observed at the periphery of nuclei in both epiblast and endoderm cells in E7.5 embryos, as we reported previously (Abe et al., 2011).

#### 2.1.3 Golgi apparatus

Both Golgi‐EGFP and Golgi‐mCherry signals in R26‐Golgi‐EGFP (Abe et al., 2011; Acc. No. CDB0246K) and R26‐Golgi‐mCherry (Abe et al., 2011; Acc. No. CDB0247K) homozygous embryos were sufficiently strong under the employed live imaging conditions. Golgi‐fluorescent signals were detected as multiple spots distributed uniformly in the cytoplasm until the early eight‐cell stage (Figure 1d,e; Supporting Information Movie S2d,e). In contrast, fluorescent signals tended to form larger clusters and accumulated around the nucleus in the blastocysts (Figure 1d,e; Supporting Information Figure S1d–e′ and Movie S2d,e). To confirm that GFP fluorescent signals represent position of Golgi apparatus, R26‐Golgi‐mCherry embryos were immuno‐stained with anti‐GM130 antibody. mCherry signals were co‐localized with immunostaining signals (Supporting Information Figure S5), suggesting that Golgi‐apparatus is visualized by Golgi‐mCherry. To analyze changes of localization more in detail, we captured time‐lapse images of Golgi‐EGFP and H2B‐mCherry reporters within shorter time windows, from the eight‐cell through blastocyst stages for up to 48 hr, in embryos obtained by the crossing between female R26‐Golgi‐EGFP and male R26‐H2B‐mCherry mice. Scattered Golgi‐EGFP signals appeared to gradually concentrate around the nucleus to form a few small clusters as embryos developed to morulae (Figure 2; Supporting Information Figure S2 and Movie S3a). Later in development, clustered fluorescent signals persisted near the nucleus through blastocyst stages, as similarly observed previously in E7.5 embryos (Abe et al., 2011). These changes in the localization of fluorescent signals were consistent with previous observations of the Golgi apparatus (Fleming & Pickering, 1985; Maro, Johnson, Pickering, & Louvard, 1985).

#### 2.1.4 Plasma membrane

Venus and mCherry gave signals along the cell membrane of blastomeres in R26‐Lyn‐Venus (Abe et al., 2011; Acc. No. CDB0254K) and R26‐Lyn‐mCherry (Acc. No. CDB0287K) homozygous embryos (Figure 1f,g; Supporting Information Figure S1f–g′ and Movie S2f,g). The intensity of Lyn‐mCherry signals appeared to be lower compared to that of Lyn‐Venus signals. However, when we used conventional laser scanning confocal microscopy, the Lyn‐mCherry signals were strong enough to delineate the cell membrane from the two‐cell stage to the blastocyst stage. In blastocysts, however, many clusters of mCherry fluorescence were also observed (Figure 1g; Supporting Information Figure S1g,g′ and Movie S1b). This finding is consistent with a previous report that mCherry tends to accumulates at the Golgi apparatus (Katayama, Yamamoto, Mizushima, Yoshimori, & Miyawaki, 2008). In both lines, some signals were also present in secretory vesicle‐like structures in the cytoplasm. Signals from Display‐mCherry (Abe et al., 2011; Acc. No. CDB0244K), Display‐Kikume Green Red (Abe et al., 2011; Acc. No. CDB0242K), and Display‐Venus (Abe et al., 2011; Acc. No. CDB0243K), which express fluorescent proteins fused with the transmembrane domain of PDGFR, were too weak to be detected along the cell membrane during preimplantation stages (data not shown), as with the previous observation at E7.5 (Abe et al., 2011).

#### 2.1.5 Microtubule

In MDCK cells, the fluorescent proteins EGFP‐Tuba (EGFP fused with alpha tubulin), hEMTB‐EGFP (EGFP fused with a tubulin binding protein EMTB), and EB1‐EGFP yielded filamentous signals (Abe et al., 2011). In KI mouse lines (Abe et al., 2011; Acc. No. CDB0245K, CDB0249K, CDB0248K, respectively), although diffuse fluorescent signals were observed in the cytoplasm in preimplantation homozygous embryos, intense signals were also observed in microtubule‐associated structures (Figure 1h–j; Supporting Information Figure S1h–j′ and Movies S1c,d,2h–j). For example, intense fluorescent signals clearly labeled mitotic spindles during mitosis in EGFP‐Tuba (Figure 3; Supporting Information Figure S3 and Movies S2h, S3e) and EB1‐EGFP embryos (Supporting Information Movie S2j). The midbody microtubules could also be observed shortly after cell division (Figure 3; Supporting Information Movies S2h, j, 3e). However, hEMTB‐EGFP did not always clearly label mitotic spindles during mitosis or midbody microtubules after cell division (Supporting Information Movie S2i).

#### 2.1.6 Actin filament

Venus‐Actin (Abe et al., 2011; Acc. No. CDB0253K) yielded weak cytoplasmic punctate signals and stronger signals along the cell periphery from the four‐cell through blastocyst stages (Figure 1k; Supporting Information Figure S1k,k′ and Movie S2k). We also tried to visualize actin filaments by using the actin‐binding domain of moesin. The fluorescent signals of Venus‐Moesin (Abe et al., 2011; Acc. No. CDB0252K) were readily and clearly detected in embryos from the two‐cell stage to the blastocyst stage. These signals were localized along the cell periphery (Figure 1l; Supporting Information Figure S1l,l′ and Movie S2l). The localizations of Venus‐Actin and Venus‐Moesin signals differed in some regions within the same cell. This difference in distribution may have arisen because Venus‐Moesin binds to a specific form of actin, such as filamentous actin, whereas Venus‐Actin also visualizes monomeric actin proteins.

#### 2.1.7 Focal adhesions

R26‐EGFP‐Paxillin (Abe et al., 2011; Acc. No. CDB0256K) was used to target fluorescent proteins to focal adhesions. EGFP‐Paxillin signals were localized uniformly in the cytoplasm from the two‐cell through eight‐cell stages. In compaction and blastocyst stage embryos, the signals manifested as small clumps over diffuse distribution in the cytoplasm (Figure 1m; Supporting Information Figure S1m,m′ and Movies S1e, S2m). We investigated whether these clumps correspond to specific structures within a cell in blastocyst stage embryos. Comparison with phalloidin staining revealed that EGFP‐Paxillin clumps localized along the phalloidin signals, suggesting that EGFP‐Paxillin binds filamentous actin (Supporting Information Figure S6).

#### 2.1.8 Tight junctions

R26‐ZO‐1‐EGFP (Katsunuma et al., 2016; Acc. No. CDB0260K), a fusion of EGFP and Zonula Occludens‐1 (ZO‐1), was used to target fluorescent proteins to tight junctions. Signals were distributed in dotted patterns in the cytoplasm, and along the cell membrane, (Figure 1n; Supporting Information Figure S1n,n′ and Movie S2n). In particular, signals appeared to be more intense at cell–cell junctions in blastocysts (Figure 1n; Supporting Information Figure S1n,n′ and Movie S1f). The ZO‐1 protein accumulates at tight junctions, where it functions as a binding protein of Claudin (Itoh et al., 1999). Tight junctions become mature by the 32‐cell stage (Eckert & Fleming, 2008; Moriwaki, Tsukita, & Furuse, 2007), and ZO‐1‐EGFP signals were observed at cell–cell junctions in these embryos. Perinuclear signals were also detected in blastocyst stage embryos, consistent with the localization of ZO‐1 described in previous reports (Fleming, Papenbrock, Fesenko, Hausen, & Sheth, 2000; Sheth et al., 2008). Although some signals were observed in the cytoplasm, sharp ZO‐1‐EGFP signals clarified the localization of ZO‐1 at tight junction as previous reports.

### 2.2 Dual labeling of different organelles

Dual labeling was performed with the following combinations of reporters: mitochondria and nucleus with R26‐Mito‐EGFP and R26‐H2B‐mCherry, Golgi apparatus and nucleus with R26‐Golgi‐EGFP and R26‐H2B‐mCherry, plasma membranes and nucleus with R26‐Lyn‐Venus and R26‐H2B‐mCherry or R26‐Lyn‐mCherry and R26‐H2B‐EGFP, and microtubule and nucleus with R26‐EGFP‐Tuba and R26‐H2B‐mCherry, respectively. In all cases, fluorescent signals derived from maternal alleles clearly marked each organelle from the two‐cell stage (Figures 2, 3, 4; Supporting Information Figure S4 and Movie S3). However, fluorescent signals derived from paternal alleles tended to be noticeably weaker at the two‐cell stage but gradually became stronger over the course of development (Supporting Information Movie S3). These results are in agreement with a previous report showing that maternal proteins are gradually degraded, whereas paternally encoded proteins accumulate through zygotic expression (Li, Zheng, & Dean, 2010). To confirm this phenomenon, we used H2B‐EGFP and H2B‐mCherry double‐heterozygous embryos generated by crossing female R26‐H2B‐EGFP and male R26‐H2B‐mCherry mice. mCherry signal intensity derived from paternal alleles gradually increased over the course of development, whereas EGFP signal intensity derived from maternal alleles did not change significantly (Supporting Information Figure S7a,b and Movie S4). In the polar body, maternal fluorescent signals were generally intense, whereas paternally encoded proteins yielded no signals. The female pronucleus was positive for green fluorescent signals throughout the period of observation, whereas the male pronucleus was initially negative but gradually became positive for red signals before the second cleavage (Supporting Information Figure S7c).

#### 2.2.1 Visualization of mitotic spindles and chromosomes during cell division

We performed live imaging of embryos heterozygous for R26‐EGFP‐Tuba and R26‐H2B‐mCherry from two‐cell and on for up to 40 hr to examine the localization of fluorescent proteins in the mitotic spindle during cell division. We observed spindle formation, visualized by EGFP‐Tubulin alpha, before cell division; chromosomes labeled with H2B‐mCherry were separated along the spindle (Figure 3; Supporting Information Movie S3e).

In addition, we examined the signals of EB1‐EGFP, which localized at the plus‐ends of extending microtubules. In one‐ and two‐cell embryos, we were able to observe EB1‐EGFP fluorescent fusion proteins moving to the plus‐ends of microtubules like comets during time‐lapse imaging (4 s interval for up to 10 min) (Supporting Information Movie S5), as reported in cultured cells (Mimori‐Kiyosue, Shiina, & Tsukita, 2000).

## 3 DISCUSSION

Live imaging is useful not only for examining cell behaviors and lineages (Kurotaki, Hatta, Nakao, Nabeshima, & Fujimori, 2007) but also for visualizing subcellular structures, including organelles and cytoskeletons. However, live imaging over the long‐term may exert toxic or disruptive effects on living cells, and such phototoxicity can disturb embryonic development (Nowotschin, Eakin, & Hadjantonakis, 2009; Yamagata et al., 2012). Many of the transgenic reporter mice established previously by conventional transgenesis carried promoters, such as CAG, to express fluorescent fusion proteins ubiquitously. However, in most cases, expression was found not always ubiquitous and showed different expression levels in different tissues (Rhee et al., 2006). In addition, high expression of fluorescent proteins is reported to cause infertility frequently (Stewart, Jang, Hong, Austin, & Behringer, 2009). In contrast, we have taken an alternative approach using gene targeting and have reported the establishment of a series of mouse lines expressing fluorescent proteins specifically localized to subcellular structures (Abe et al., 2011; Katsunuma et al., 2016; Shioi et al., 2011). These reporter mouse lines could circumvent common problems in other transgenic reporter mice as mentioned above. Indeed, our R26 reporter mouse lines have proven very useful for live cell imaging of not only early embryos (Abe et al., 2011; Ichikawa et al., 2013; Kawaue et al., 2014; Ke et al., 2016; Lau et al., 2015; Namba et al., 2014; Nonomura et al., 2013; Okamoto et al., 2013; Shimozawa et al., 2013; Shioi et al., 2017), but also several of adult tissue types and primary cultured cells obtained from these mice (Ito et al., 2018; Jin et al., 2017; Shinomura et al., 2014; Son et al., 2011; Susaki et al., 2014; Tainaka et al., 2014; Yokose et al., 2017).

The current study also demonstrated that most of the reporter mouse lines we have examined are suitable for long‐term live cell imaging from two‐cell through blastocyst stages without apparent negative effects on preimplantation embryo development. The total cell number of blastocyst embryos was comparable to that of non‐imaged and wild‐type embryos. Cell shape and cytoskeleton were readily visualized with their dynamics successfully recorded over the course of development. In particular, without live imaging, it is generally difficult to understand the dynamics of organelle such as mitochondria and Golgi apparatus, which change localization dramatically during preimplantation stages. We could continuously observe changes in distributions of fluorescently labeled proteins localized to the mitochondria and Golgi apparatus, and their localizations patterns were consistent with the shapes and positions of these organelles in fixed embryos (Basu et al., 2008; Fleming & Pickering, 1985; Kawamura et al., 2005; Lee et al., 2011; Maro et al., 1985). To our knowledge, this is the first report to show the changes in the localization of Golgi apparatus continuously and appearance of mitochondria in preimplantation mouse embryos by live imaging.

Fluorescent signals from the reporter alleles were strong enough for live imaging, regardless of gene dosage; that is, all heterozygous embryos, as well as homozygotes, yielded detectable signals. In addition, embryos derived from zygotes generated via in vitro fertilization, and once cryopreserved were also amenable to live imaging without noticeable loss of quality and viability. Fluorescent signals differed when reporter genes were transmitted from maternal or paternal alleles, as revealed by an apparent difference in H2B fluorescent signal intensities in R26‐H2B‐EGFP and R26‐H2B‐mCherry double heterozygous embryos. The female pronucleus and second polar body were labeled with maternal H2B fluorescent proteins after fertilization. The male pronucleus also acquired maternal fluorescent proteins before the first round of cell division. Accordingly, maternal proteins exhibited no significant changes in signal intensities during development. In contrast, paternal proteins gradually accumulated during the two‐cell and four‐cell stages. This phenomenon is consistent with previous reports to suggest that early mammalian embryogenesis is almost completely dependent on the egg for the initial supply of subcellular organelles and macromolecules (Li et al., 2010), and that zygotic gene expression starts from the late one‐cell stage (Hamatani, Carter, Sharov, & Ko, 2004).

This study also provided an example of side‐by‐side comparison of the performance of EGFP versus Venus as fluorescent probes. Venus tends to yield brighter signals than EGFP, but its photostability is shown to be substantially lower (Shaner, Steinbach, & Tsien, 2005). With both types of probes, we could achieve enough fluorescent signal to perform live imaging from the two‐cell through blastocyst stages. However, Venus yielded more specific membrane signals than EGFP when fused with the myristoylation signal of Lyn or the actin‐binding sequence of moesin (data not shown), although identical sequences for fusion were used, prompting us to favor Venus for these probes.

In cultured mammalian cells, the Golgi apparatus undergoes extensive fragmentation during cell division and is subsequently reconstituted (Lucocq, Pryde, Berger, & Warren, 1987; Robbins & Gonatas, 1964). However, in our experiments, we observed somewhat unexpected distribution patterns of fluorescent signals in preimplantation embryos. In both Golgi‐EGFP and Golgi‐mCherry embryos, distribution of fluorescent signals changed from diffuse cytoplasmic to localized perinuclear appearances over the course of embryonic development (Figure 2, Supporting Information Movie S3a). Such changes appear to corroborate previous observations made in fixed samples of preimplantation embryos (Maro et al., 1985; Fleming and Pickering., 1985). However, we could not observe the reported localization pattern of the Golgi apparatus in the basal cytoplasm from the compaction stage to the blastocyst stage.

To visualize microtubules in developing embryos, we examined three types of fluorescent proteins, hEMTB‐EGFP, EGFP‐Tuba, and EB1‐EGFP. hEMTB‐EGFP signals were more intense on apical region at the compaction stage, as described previously (Supporting Information Movie S2i; Kono, Tamashiro, & Alarcon, 2014), possibly because the microtubule network is concentrated in the apical domain (Lechler & Fuchs, 2007). However, this phenomenon was not observed at other stages or in EGFP‐Tuba and EB1‐EGFP embryos (Supporting Information Movie S2h–j). In contrast, condensing spindle microtubules at M‐phase were detected in EGFP‐Tuba and EB1‐EGFP embryos, but not in hEMTB‐EGFP embryos. In addition, midbody microtubules were detected in early stages of EGFP‐Tuba and EB1‐EGFP embryos but were only detected in the late stages in hEMTB‐EGFP embryos. The underlying causes of these differences would be of further interest worth pursued in future studies.

We also observed fluorescent comets on the mitotic spindles in EB1‐EGFP‐expressing embryos during cell division (Supporting Information Movie S5). Considering the long exposure period, the shapes of these comets may reflect the speed of movement of the plus‐ends of microtubules. The employed conditions failed to capture images to reconstitute completely continuous sequence of events, while imaging in shorter intervals could have resulted in phototoxicity.

Most mouse lines were suitable for live imaging using homozygous embryos, with the exception of Venus‐Actin. Homozygous Venus‐Actin mice were rarely obtained after mating of heterozygous mice. Moreover, even when heterozygous female mice were mated with wild‐type male mice, we could not obtain the expected number of heterozygous mice. Therefore, we only used heterozygous Venus‐Actin embryos derived from male mice carrying the reporter gene. This observation suggests that embryonic development was somehow impaired by maternal Venus‐Actin protein accumulated in oocytes or in embryos. Given that this occurred without exposure to light, it suggests that expression of Venus‐Actin per se was toxic to embryos.

Based on the observations discussed above, we conclude that most of the mouse lines that we subjected to live imaging during preimplantation development are useful for visualizing specific subcellular structures in developing embryos. In this study, we used mice that had no other gene alterations. Plausibly, the reporter mice described here can be combined with various other modified genetic backgrounds; for example, by crossing with mutant lines deficient for specific genes. This type of approaches would be instrumental to investigate whether a given gene of interest plays a role in regulating features of cytoskeletal dynamics, chromosome segregation or organelle behaviors.

## 4 MATERIALS AND METHODS

### 4.1 Mouse lines for live imaging

Mouse lines ubiquitously expressing fluorescent fusion proteins (Abe et al., 2011; Katsunuma et al., 2016) under the control of the endogenous R26 promoter were used to obtain embryos for live imaging. In addition, to visualize the plasma membrane, we generated a new mouse line expressing a Lyn‐mCherry fusion protein, as described previously (Abe et al., 2011). The R26‐Lyn‐mCherry (Acc. No. CDB0287K) line, which lacks the neo cassette and ubiquitously expresses the fluorescent fusion protein, was generated by crossing homozygous R26R‐Lyn‐mCherry (Acc. No. CDB0278K) mice with Ella‐Cre transgenic mice (Lakso et al., 1996). These mouse lines will be available to the research community (http://www2.clst.riken.jp/arg/reporter_mice.html). All animal experiments were approved by the Institutional Animal Care and Use Committees (IACUCs) of RIKEN Kobe Branch and the National Institutes of Natural Sciences.

### 4.2 Embryo culture

Homozygous and heterozygous embryos, except for pronuclear stage embryos, were prepared by in vitro fertilization (IVF) and frozen at the two‐cell stage. After thawing, these embryos were cultured in KSOM medium (ARK Resource) from the two‐cell stage to the blastocyst stage. Pronuclear stage embryos were freshly obtained from oviducts and immediately used for analyses.

### 4.3 Live imaging of mouse embryo

Time‐lapse images of embryos from two‐cell through blastocyst stages were obtained with the Olympus LCV100 incubation imaging system equipped with a spinning confocal system, CSU10 (Yokogawa), and the iXon+EMCCD camera (Andor). Live images of H2B‐EGFP × H2B‐mCherry embryos during the one‐cell stage were obtained with a spinning disc confocal imaging system CV1000 (Yokogawa). The embryos were cultured in 5% CO_2_ at 37°C; a 488‐nm laser was used to acquire EGFP and Venus images, and a 561‐nm laser was used to acquire mCherry images. Images of embryos from the two‐cell through blastocyst stages were taken every hour for up to 72 hr with 19 Z‐sections at 5 μm intervals were acquired at each time point. Image data were analyzed using the MetaMorph software (Universal Imaging Corporation). Images of Golgi‐EGFP × H2B‐mCherry embryos from the eight‐cell through blastocyst stages and EGFP‐Tuba × H2B‐mCherry embryos from the two‐cell through morula stages were taken every 2 min for up to 48 and 40 hr, respectively, with 19 Z‐sections at 5 μm intervals acquired at each time point. Images of H2B‐EGFP × H2B‐mCherry embryos during the one‐cell stage were taken every 10 min with 13 Z‐sections at 5 μm intervals acquired at each time point. To examine the behavior of comets of microtubule plus‐ends, embryos‐expressing EB1‐EGFP were cultured in the MIGM Incubator System for Microscopes (Tokken), and time‐lapse images were acquired on the Nikon Ti‐s A1 confocal microscope. Live images of embryos were taken every 4 s for up to 10 min. Image data were analyzed using the MetaMorph software.

### 4.4 Observation of fine structures of embryos

To visualize nuclei, embryos were incubated with Hoechst 33342 (Invitrogen) in KSOM medium at 37°C with 5% CO_2_ for 30 min without fixation. EGFP‐Paxillin embryos were fixed with 4% PFA in PBS for 20 min at room temperature and then stained with rhodamine–phalloidin (Invitrogen) at room temperature for 30 min to visualize f‐actin. Golgi‐mCherry embryos were fixed with 4% PFA in PBS for 20 min at room temperature, and then Golgi apparatus was stained with mouse anti GM130 antibody (1:200, BD Biosciences, 610822) and an Alexa‐488‐conjugated secondary antibody (1:500, Invitrogen Co., Ltd). DAPI (nacalai tesque) was used as nuclear counterstain. Embryos were imaged on a Nikon A1‐Ti confocal microscope using a ×40/0.95 NA objective lens at the two‐cell, four‐cell, eight‐cell, and blastocyst stages.

### 4.5 Cell counting of blastocyst embryos

Total cell number of blastocyst stage embryos (66 hr after thawing) was counted following Hoechst 33342 staining and imaging using a Nikon A1‐Ti confocal microscope. The averages of cell number were calculated from five embryos (39, 36, 39, 33, and 46 cells) for the time‐lapse imaged, eight embryos (40, 32, 38, 42, 38, 38, 35, and 40 cells) for non‐imaged, and five embryos (42, 31, 38, 32, and 30 cells) for wild‐type, respectively.

## Supporting information


**Supporting information Figure 1 Expression of fluorescent fusion proteins in blastocysts .** (a, a’) H2B‐EGFP, (b, b’) H2B‐mCherry, (c, c’) Mito‐EGFP, (d, d’) Golgi‐EGFP, (e, e’) Golgi‐mCherry, (f, f’) Lyn‐Venus, (g, g’) Lyn‐mCherry, (h, h’) EGFP‐Tuba, (i, i’) hEMTB‐EGFP, (j, j’) EB1‐EGFP, (k, k’) Venus‐Actin, (l, l’) Venus‐Moesin, (m, m’) EGFP‐Paxillin, and (n, n’) ZO1‐EGFP. (a‐n) images represent single Z‐sections from blastocyst shown in Figure 1, (a’‐n’) enlarged views of the areas boxed in (a‐n) without nuclear DAPI staining. Scale bar = 20 μm (a‐n), 5 μm (a’‐n’).

**Supporting information Figure 2 Expression of fluorescent fusion proteins in 8‐cell R26‐Golgi‐EGFP/H2B‐mCherry embryos.** Golgi‐EGFP/H2B‐mCherry (left), H2B‐mCherry (middle) and Golgi‐EGFP (right). The left panel represents a single Z‐section from Figure 2, and the middle and right panels are enlarged views of the area boxed in the left panel. Scale bar = 20 μm (left), 5 μm (middle and right).

**Supporting information Figure 3 Expression of fluorescent fusion protein in 2‐cell R26‐EGFP‐Tuba/H2B‐mCherry embryos.** EGFP‐Tuba/H2B‐mCherry (left), H2B‐mCherry (middle) and EGFP‐Tuba (right). The left panel represents a single Z‐section from Figure 3. The middle and left panels are enlarged views of the area boxed in the left panel. Scale bar = 20 μm (left), 10 μm (middle and right).

**Supporting information Figure 4 Expression of fluorescent fusion proteins in dual‐labeled blastocysts.** (a) Mito‐EGFP/H2B‐mCherry, (a’) H2B‐mCherry, (a”) Mito‐EGFP, (b) Golgi‐EGFP/H2B‐mCherry, (b’) H2B‐mCherry, (b”) Golgi‐EGFP, (c) Lyn‐Venus/H2B‐mCherry, (c’) H2B‐mCherry, (c”) Lyn‐Venus, (d) Lyn‐mCherry/H2B‐EGFP, (d’) H2B‐EGFP, (d”) Lyn‐mCherry, (e) EGFP‐Tuba/H2B‐mCherry, (e’) H2B‐mCherry, (e”) EGFP‐Tuba. (a‐e) the images represent single Z‐sections from the blastocysts in Figure 4. (a’‐e’, a”‐e”) enlarged views of the areas boxed in (a‐e). Scale bar = 20 μm (a‐e), 10 μm (a’‐e’, a”‐e”).

**Supporting information Figure 5 Immunostaining of Golgi apparatus in a Golgi‐mCherry blastocysts.** (a) Immunostaining for Golgi apparatus. (b) Golgi‐mCherry fluorescent fusion protein. (c) merged image. (c’) an enlarged view of the area boxed in (c). Scale bar = 20 μm (a‐c), 5 μm (c’).

**Supporting information Figure 6 Expression of the fluorescent fusion protein in the blastocysts expressing R26‐EGFP‐Paxillin.** (a, d) EGFP‐Paxillin. (b, e) phalloidin staining. (c, f) merged image. (c’, c”) enlarged views of the area boxed in (c). (f’, f”) enlarged views of the area boxed in (f). Images in (a–c) and (d–f) are MIPs. Scale bar = 20 μm (a–c and d–f), 5 μm (c’, c”, f’, and f”).

**Supporting information Figure 7 Changes of signal intensities in nuclei during pre‐implantation development.** (a) Time‐lapse images of an embryo obtained by crossing female R26‐H2B‐EGFP and male R26‐H2B‐mCherry mice developing from the 2‐cell through blastocyst stages. Time‐lapse images were taken every 1 hour; selected images at 5‐hour intervals (i.e., every five images) are shown. The z‐step size is 5 μm. Scale bar = 20 μm. (b) Analysis of signal intensities of H2B‐mCherry and H2B‐EGFP from time‐lapse images in (a). Green solid and dotted lines indicate EGFP signal intensities of different blastomeres in the same embryo. Red solid and dotted lines indicate mCherry signal intensities of different blastomeres in the same embryo. The intensity of each signal at 25 hours was defined as 1.0, and each time point is expressed as a relative value. (c) Time‐lapse images of a developing embryo obtained by crossing R26‐H2B‐mCherry and R26‐H2B‐EGFP during the pronuclear stage. Embryos were obtained from the oviducts. Time‐lapse images were taken every 10 min; selected images at 2‐hour intervals (i.e., every 12 images) are shown. Time stamps are shown on the bottom. H2B‐mCherry and H2B‐EGFP images are MIPs. The z‐step size is 5 μm. Scale bar = 20 μm.

**Supporting information movie 1 Movies of images acquired after changing the z positions of reporter mouse embryos.** (a) Mito‐EGFP, (b) Lyn‐mCherry, (c) hEMTB‐EGFP, (d) EB1‐EGFP, (e) EGFP‐Paxillin, and (f) ZO1‐EGFP. (a), (b), (e) and (f) are blastocyst stages; (c), 2‐cell stage; and (d), 8‐cell stage. The z‐step size is 2.5 μm.

**Supporting information movie 2 Time‐lapse imaging of reporter mouse embryos from the 2‐cell through blastocyst stages.** (a) H2B‐EGFP, (b) H2B‐mCherry, (c) Mito‐EGFP, (d) Golgi‐EGFP, (e) Golgi‐mCherry, (f) Lyn‐Venus, (g) Lyn‐mCherry, (h) EGFP‐Tuba, (i) hEMTB‐EGFP, (j) EB1‐EGFP, (k) Venus‐Actin, (l) Venus‐Moesin, (m) EGFP‐Paxillin, and (n) ZO1‐EGFP. Time interval is 1 hour. The z‐step size is 2.5 μm. Left, DIC images; right, fluorescent MIP images.

**Supporting information movie 3 Time‐lapse imaging in dual‐labeled embryos.** Embryos were obtained by crossing (a) R26‐Golgi‐EGFP and R26‐H2B‐mCherry, (b) R26‐Mito‐EGFP and R26‐H2B‐mCherry, (c) R26‐Lyn‐Venus and R26‐H2B‐mCherry, (d) R26‐Lyn‐mCherry and R26‐H2B‐EGFP, and (e) R26‐EGFP‐Tuba and R26‐H2B‐mCherry, respectively. (a) is from the 8‐cell through blastocyst stages; (b), (c), and (d) are from the 2‐cell through blastocyst stages; and (e) is from the 2‐cell through 4‐cell stages. Time intervals are 2 min for (a) and (e), and 1 hour for (b), (c), and (d). The z‐step size is 2.5 μm. Top left, DIC image; top right, merged MIP image; bottom left, H2B‐fluorescent MIP image; bottom right, MIP images of each fluorescent color.

**Supporting information movie 4 Time‐lapse imaging of changes in signal intensities in the nuclei.** Embryos were obtained by crossing R26‐H2B‐EGFP and R26‐H2B‐mCherry. Time‐lapse images are from the 2‐cell through blastocyst stages. Time interval is 1 hour. The z‐step size is 5 μm. Top left, DIC image; top right, H2B‐EGFP MIP image; bottom left, H2B‐mCherry MIP image.

**Supporting information movie 5 Comet‐like signals in EB1‐EGFP embryos.** Time‐lapse images in 1‐cell and 2‐cell homozygous R26‐EB1‐EGFP embryos. Nuclei were stained with Hoechst 33342 (blue). (a) First round of cell division after the 1‐cell stage. (b) Division at the 2‐cell stage. Time interval is 4 seconds.

## References

[dvg23277-bib-0001] Abe, T. , & Fujimori, T. (2013). Reporter mouse lines for fluorescence imaging. Development, Growth & Differentiation, 55(4), 390–405. 10.1111/dgd.12062 23621623

[dvg23277-bib-0002] Abe, T. , Kiyonari, H. , Shioi, G. , Inoue, K. , Nakao, K. , Aizawa, S. , & Fujimori, T. (2011). Establishment of conditional reporter mouse lines at ROSA26 locus for live cell imaging. Genesis, 49(7), 579–590. 10.1002/dvg.20753 21445964

[dvg23277-bib-0003] Basu, B. , Desai, R. , Balaji, J. , Chaerkady, R. , Sriram, V. , Maiti, S. , & Panicker, M. M. (2008). Serotonin in pre‐implantation mouse embryos is localized to the mitochondria and can modulate mitochondrial potential. Reproduction, 135(5), 657–669. 10.1530/REP-07-0577 18304982

[dvg23277-bib-0004] Bevis, B. J. , & Glick, B. S. (2002). Rapidly maturing variants of the discosoma red fluorescent protein (DsRed). Nature Biotechnology, 20(1), 83–87. 10.1038/nbt0102-83 11753367

[dvg23277-bib-0005] Bulina, M. E. , Lukyanov, K. A. , Britanova, O. V. , Onichtchouk, D. , Lukyanov, S. , & Chudakov, D. M. (2006). Chromophore‐assisted light inactivation (CALI) using the phototoxic fluorescent protein KillerRed. Nature Protocols, 1(2), 947–953. 10.1038/nprot.2006.89 17406328

[dvg23277-bib-0006] Chalfie, M. , Tu, Y. , Euskirchen, G. , Ward, W. W. , & Prasher, D. C. (1994). Green fluorescent protein as a marker for gene expression. Science, 263(5148), 802–805. 8303295 10.1126/science.8303295

[dvg23277-bib-0007] Chudakov, D. M. , Matz, M. V. , Lukyanov, S. , & Lukyanov, K. A. (2010). Fluorescent proteins and their applications in imaging living cells and tissues. Physiological Reviews, 90(3), 1103–1163. 10.1152/physrev.00038.2009 20664080

[dvg23277-bib-0008] Eckert, J. J. , & Fleming, T. P. (2008). Tight junction biogenesis during early development. Biochimica et Biophysica Acta, 1778(3), 717–728. 10.1016/j.bbamem.2007.09.031 18339299

[dvg23277-bib-0009] Fleming, T. P. , & Pickering, S. J. (1985). Maturation and polarization of the endocytotic system in outside blastomeres during mouse preimplantation development. Journal of Embryology and Experimental Morphology, 89, 175–208. 4093747

[dvg23277-bib-0010] Fleming, T. P. , Papenbrock, T. , Fesenko, I. , Hausen, P. , & Sheth, B. (2000). Assembly of tight junctions during early vertebrate development. Seminars in Cell & Developmental Biology, 11(4), 291–299. 10.1006/scdb.2000.0179 10966863

[dvg23277-bib-0011] Hamatani, T. , Carter, M. G. , Sharov, A. A. , & Ko, M. S. (2004). Dynamics of global gene expression changes during mouse preimplantation development. Developmental Cell, 6(1), 117–131. 14723852 10.1016/s1534-5807(03)00373-3

[dvg23277-bib-0012] Hoshino, H. , Nakajima, Y. , & Ohmiya, Y. (2007). Luciferase‐YFP fusion tag with enhanced emission for single‐cell luminescence imaging. Nature Methods, 4(8), 637–639. 10.1038/nmeth1069 17618293

[dvg23277-bib-0013] Ichikawa, T. , Nakazato, K. , Keller, P. J. , Kajiura‐Kobayashi, H. , Stelzer, E. H. , Mochizuki, A. , & Nonaka, S. (2013). Live imaging of whole mouse embryos during gastrulation: Migration analyses of epiblast and mesodermal cells. PLoS One, 8(7), e64506 10.1371/journal.pone.0064506 23861733 PMC3704669

[dvg23277-bib-0014] Ito, N. , Katoh, K. , Kushige, H. , Saito, Y. , Umemoto, T. , Matsuzaki, Y. , … Ohta, K. (2018). Ribosome incorporation into somatic cells promotes lineage transdifferentiation towards multipotency. Scientific Reports, 8(1), 1634 10.1038/s41598-018-20057-1 29374279 PMC5786109

[dvg23277-bib-0015] Itoh, M. , Furuse, M. , Morita, K. , Kubota, K. , Saitou, M. , & Tsukita, S. (1999). Direct binding of three tight junction‐associated MAGUKs, ZO‐1, ZO‐2, and ZO‐3, with the COOH termini of claudins. The Journal of Cell Biology, 147(6), 1351–1363. 10601346 10.1083/jcb.147.6.1351 PMC2168087

[dvg23277-bib-0016] Jin, Y. , Muhl, L. , Burmakin, M. , Wang, Y. , Duchez, A. C. , Betsholtz, C. , … Jakobsson, L. (2017). Endoglin prevents vascular malformation by regulating flow‐induced cell migration and specification through VEGFR2 signalling. Nature Cell Biology, 19(6), 639–652. 10.1038/ncb3534 28530660 PMC5467724

[dvg23277-bib-0017] Katayama, H. , Yamamoto, A. , Mizushima, N. , Yoshimori, T. , & Miyawaki, A. (2008). GFP‐like proteins stably accumulate in lysosomes. Cell Structure and Function, 33(1), 1–12. 18256512 10.1247/csf.07011

[dvg23277-bib-0018] Katsunuma, S. , Honda, H. , Shinoda, T. , Ishimoto, Y. , Miyata, T. , Kiyonari, H. , … Togashi, H. (2016). Synergistic action of nectins and cadherins generates the mosaic cellular pattern of the olfactory epithelium. The Journal of Cell Biology, 212(5), 561–575. 10.1083/jcb.201509020 26929452 PMC4772500

[dvg23277-bib-0019] Kawamura, K. , Fukuda, J. , Kumagai, J. , Shimizu, Y. , Kodama, H. , Nakamura, A. , & Tanaka, T. (2005). Gonadotropin‐releasing hormone I analog acts as an antiapoptotic factor in mouse blastocysts. Endocrinology, 146(9), 4105–4116. 10.1210/en.2004-1646 15932933

[dvg23277-bib-0020] Kawaue, T. , Sagou, K. , Kiyonari, H. , Ota, K. , Okamoto, M. , Shinoda, T. , … Miyata, T. (2014). Neurogenin2‐d4Venus and Gadd45g‐d4Venus transgenic mice: Visualizing mitotic and migratory behaviors of cells committed to the neuronal lineage in the developing mammalian brain. Development, Growth & Differentiation, 56(4), 293–304. 10.1111/dgd.12131 PMC4477914 24712911

[dvg23277-bib-0021] Ke, M. T. , Nakai, Y. , Fujimoto, S. , Takayama, R. , Yoshida, S. , Kitajima, T. S. , … Imai, T. (2016). Super‐resolution mapping of neuronal circuitry with an index‐optimized clearing agent. Cell Reports, 14(11), 2718–2732. 10.1016/j.celrep.2016.02.057 26972009

[dvg23277-bib-0022] Kono, K. , Tamashiro, D. A. , & Alarcon, V. B. (2014). Inhibition of RHO‐ROCK signaling enhances ICM and suppresses TE characteristics through activation of Hippo signaling in the mouse blastocyst. Developmental Biology, 394(1), 142–155. 10.1016/j.ydbio.2014.06.023 24997360 PMC4404313

[dvg23277-bib-0023] Kurotaki, Y. , Hatta, K. , Nakao, K. , Nabeshima, Y. , & Fujimori, T. (2007). Blastocyst axis is specified independently of early cell lineage but aligns with the ZP shape. Science, 316(5825), 719–723. 10.1126/science.1138591 17446354

[dvg23277-bib-0024] Lakso, M. , Pichel, J. G. , Gorman, J. R. , Sauer, B. , Okamoto, Y. , Lee, E. , … Westphal, H. (1996). Efficient in vivo manipulation of mouse genomic sequences at the zygote stage. Proceedings of the National Academy of Sciences of the United States of America, 93(12), 5860–5865. 8650183 10.1073/pnas.93.12.5860 PMC39152

[dvg23277-bib-0025] Lau, K. , Tao, H. , Liu, H. , Wen, J. , Sturgeon, K. , Sorfazlian, N. , … Hopyan, S. (2015). Anisotropic stress orients remodelling of mammalian limb bud ectoderm. Nature Cell Biology, 17(5), 569–579. 10.1038/ncb3156 25893915 PMC4955842

[dvg23277-bib-0026] Lechler, T. , & Fuchs, E. (2007). Desmoplakin: An unexpected regulator of microtubule organization in the epidermis. The Journal of Cell Biology, 176(2), 147–154. 10.1083/jcb.200609109 17227889 PMC2063934

[dvg23277-bib-0027] Lee, S. E. , Hwang, K. C. , Sun, S. C. , Xu, Y. N. , & Kim, N. H. (2011). Modulation of autophagy influences development and apoptosis in mouse embryos developing in vitro. Molecular Reproduction and Development, 78(7), 498–509. 10.1002/mrd.21331 21681844

[dvg23277-bib-0028] Li, L. , Zheng, P. , & Dean, J. (2010). Maternal control of early mouse development. Development, 137(6), 859–870. 10.1242/dev.039487 20179092 PMC2834456

[dvg23277-bib-0029] Lucocq, J. M. , Pryde, J. G. , Berger, E. G. , & Warren, G. (1987). A mitotic form of the Golgi apparatus in HeLa cells. The Journal of Cell Biology, 104(4), 865–874. 3104351 10.1083/jcb.104.4.865 PMC2114436

[dvg23277-bib-0030] Maro, B. , Johnson, M. H. , Pickering, S. J. , & Louvard, D. (1985). Changes in the distribution of membranous organelles during mouse early development. Journal of Embryology and Experimental Morphology, 90, 287–309. 3834033

[dvg23277-bib-0031] Mimori‐Kiyosue, Y. , Shiina, N. , & Tsukita, S. (2000). The dynamic behavior of the APC‐binding protein EB1 on the distal ends of microtubules. Current Biology, 10(14), 865–868. 10899006 10.1016/s0960-9822(00)00600-x

[dvg23277-bib-0032] Moriwaki, K. , Tsukita, S. , & Furuse, M. (2007). Tight junctions containing claudin 4 and 6 are essential for blastocyst formation in preimplantation mouse embryos. Developmental Biology, 312(2), 509–522. 10.1016/j.ydbio.2007.09.049 17980358

[dvg23277-bib-0033] Namba, T. , Kibe, Y. , Funahashi, Y. , Nakamuta, S. , Takano, T. , Ueno, T. , … Kaibuchi, K. (2014). Pioneering axons regulate neuronal polarization in the developing cerebral cortex. Neuron, 81(4), 814–829. 10.1016/j.neuron.2013.12.015 24559674

[dvg23277-bib-0034] Nonomura, K. , Yamaguchi, Y. , Hamachi, M. , Koike, M. , Uchiyama, Y. , Nakazato, K. , … Miura, M. (2013). Local apoptosis modulates early mammalian brain development through the elimination of morphogen‐producing cells. Developmental Cell, 27(6), 621–634. 10.1016/j.devcel.2013.11.015 24369835

[dvg23277-bib-0035] Nowotschin, S. , Eakin, G. S. , & Hadjantonakis, A. K. (2009). Live‐imaging fluorescent proteins in mouse embryos: Multi‐dimensional, multi‐spectral perspectives. Trends in Biotechnology, 27(5), 266–276. 10.1016/j.tibtech.2009.02.006 19339068 PMC2878313

[dvg23277-bib-0036] Okamoto, M. , Namba, T. , Shinoda, T. , Kondo, T. , Watanabe, T. , Inoue, Y. , … Miyata, T. (2013). TAG‐1‐assisted progenitor elongation streamlines nuclear migration to optimize subapical crowding. Nature Neuroscience, 16(11), 1556–1566. 10.1038/nn.3525 24056697

[dvg23277-bib-0037] Rhee, J. M. , Pirity, M. K. , Lackan, C. S. , Long, J. Z. , Kondoh, G. , Takeda, J. , & Hadjantonakis, A. K. (2006). In vivo imaging and differential localization of lipid‐modified GFP‐variant fusions in embryonic stem cells and mice. Genesis, 44(4), 202–218. 10.1002/dvg.20203 16604528 PMC2887760

[dvg23277-bib-0038] Robbins, E. , & Gonatas, N. K. (1964). The ultrastructure of a mammalian cell during the mitotic cycle. The Journal of Cell Biology, 21, 429–463. 14189913 10.1083/jcb.21.3.429 PMC2106374

[dvg23277-bib-0039] Shaner, N. C. , Campbell, R. E. , Steinbach, P. A. , Giepmans, B. N. , Palmer, A. E. , & Tsien, R. Y. (2004). Improved monomeric red, orange and yellow fluorescent proteins derived from Discosoma sp. red fluorescent protein. Nature Biotechnology, 22(12), 1567–1572. 10.1038/nbt1037 15558047

[dvg23277-bib-0040] Shaner, N. C. , Steinbach, P. A. , & Tsien, R. Y. (2005). A guide to choosing fluorescent proteins. Nature Methods, 2(12), 905–909. 10.1038/nmeth819 16299475

[dvg23277-bib-0041] Sheth, B. , Nowak, R. L. , Anderson, R. , Kwong, W. Y. , Papenbrock, T. , & Fleming, T. P. (2008). Tight junction protein ZO‐2 expression and relative function of ZO‐1 and ZO‐2 during mouse blastocyst formation. Experimental Cell Research, 314(18), 3356–3368. 10.1016/j.yexcr.2008.08.021 18817772

[dvg23277-bib-0042] Shimomura, O. , Johnson, F. H. , & Saiga, Y. (1962). Extraction, purification and properties of aequorin, a bioluminescent protein from the luminous hydromedusan, Aequorea. Journal of Cellular and Comparative Physiology, 59, 223–239. 13911999 10.1002/jcp.1030590302

[dvg23277-bib-0043] Shimozawa, T. , Yamagata, K. , Kondo, T. , Hayashi, S. , Shitamukai, A. , Konno, D. , … Mimori‐Kiyosue, Y. (2013). Improving spinning disk confocal microscopy by preventing pinhole cross‐talk for intravital imaging. Proceedings of the National Academy of Sciences of the United States of America, 110(9), 3399–3404. 10.1073/pnas.1216696110 23401517 PMC3587224

[dvg23277-bib-0044] Shinomura, M. , Kishi, K. , Tomita, A. , Kawasumi, M. , Kanezashi, H. , Kuroda, Y. , … Kanai, Y. (2014). A novel Amh‐Treck transgenic mouse line allows toxin‐dependent loss of supporting cells in gonads. Reproduction, 148(6), H1–H9. 10.1530/REP-14-0171 25212783

[dvg23277-bib-0045] Shioi, G. , Kiyonari, H. , Abe, T. , Nakao, K. , Fujimori, T. , Jang, C. W. , … Aizawa, S. (2011). A mouse reporter line to conditionally mark nuclei and cell membranes for in vivo live‐imaging. Genesis, 49(7), 570–578. 10.1002/dvg.20758 21504045 PMC3466090

[dvg23277-bib-0046] Shioi, G. , Hoshino, H. , Abe, T. , Kiyonari, H. , Nakao, K. , Meng, W. , … Aizawa, S. (2017). Apical constriction in distal visceral endoderm cells initiates global, collective cell rearrangement in embryonic visceral endoderm to form anterior visceral endoderm. Developmental Biology, 429(1), 20–30. 10.1016/j.ydbio.2017.07.004 28712875

[dvg23277-bib-0047] Son, E. Y. , Ichida, J. K. , Wainger, B. J. , Toma, J. S. , Rafuse, V. F. , Woolf, C. J. , & Eggan, K. (2011). Conversion of mouse and human fibroblasts into functional spinal motor neurons. Cell Stem Cell, 9(3), 205–218. 10.1016/j.stem.2011.07.014 21852222 PMC3188987

[dvg23277-bib-0048] Stewart, M. D. , Jang, C. W. , Hong, N. W. , Austin, A. P. , & Behringer, R. R. (2009). Dual fluorescent protein reporters for studying cell behaviors in vivo. Genesis, 47(10), 708–717. 10.1002/dvg.20565 19813259 PMC3384709

[dvg23277-bib-0049] Susaki, E. A. , Tainaka, K. , Perrin, D. , Kishino, F. , Tawara, T. , Watanabe, T. M. , … Ueda, H. R. (2014). Whole‐brain imaging with single‐cell resolution using chemical cocktails and computational analysis. Cell, 157(3), 726–739. 10.1016/j.cell.2014.03.042 24746791

[dvg23277-bib-0050] Tainaka, K. , Kubota, S. I. , Suyama, T. Q. , Susaki, E. A. , Perrin, D. , Ukai‐Tadenuma, M. , … Ueda, H. R. (2014). Whole‐body imaging with single‐cell resolution by tissue decolorization. Cell, 159(4), 911–924. 10.1016/j.cell.2014.10.034 25417165

[dvg23277-bib-0051] Trichas, G. , Begbie, J. , & Srinivas, S. (2008). Use of the viral 2A peptide for bicistronic expression in transgenic mice. BMC Biology, 6, 40 10.1186/1741-7007-6-40 18793381 PMC2553761

[dvg23277-bib-0052] Yamagata, K. , Iwamoto, D. , Terashita, Y. , Li, C. , Wakayama, S. , Hayashi‐Takanaka, Y. , … Wakayama, T. (2012). Fluorescence cell imaging and manipulation using conventional halogen lamp microscopy. PLoS One, 7(2), e31638 10.1371/journal.pone.0031638 22347500 PMC3275630

[dvg23277-bib-0053] Yokose, J. , Okubo‐Suzuki, R. , Nomoto, M. , Ohkawa, N. , Nishizono, H. , Suzuki, A. , … Inokuchi, K. (2017). Overlapping memory trace indispensable for linking, but not recalling, individual memories. Science, 355(6323), 398–403. 10.1126/science.aal2690 28126819

[dvg23277-bib-0054] Zhang, G. , Gurtu, V. , & Kain, S. R. (1996). An enhanced green fluorescent protein allows sensitive detection of gene transfer in mammalian cells. Biochemical and Biophysical Research Communications, 227(3), 707–711. 10.1006/bbrc.1996.1573 8885998

