# Supplementary figures and images for: Dynamic organelle localization and cytoskeletal reorganization during preimplantation mouse embryo development revealed by live imaging of genetically encoded fluorescent fusion proteins
Source: Genesis. 2019 Jan 13;57(2):e23277. doi: 10.1002/dvg.23277 (PMC6590263; doi:10.1002/dvg.23277)

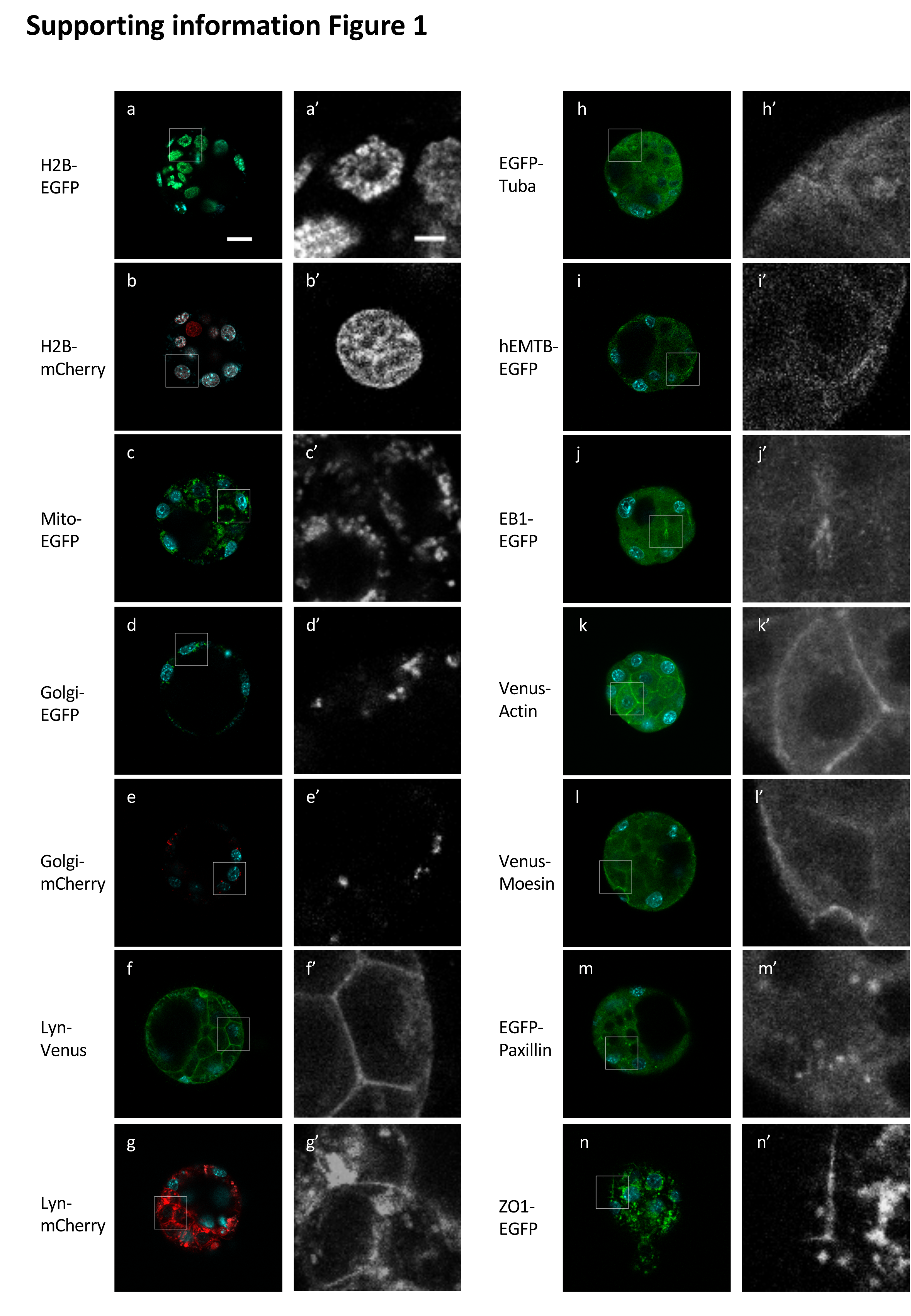

Supplement: Supplementary file 1 — Supporting information Figure 1 Expression of fluorescent fusion proteins in blastocysts . (a, a’) H2B‐EGFP, (b, b’) H2B‐mCherry, (c, c’) Mito‐EGFP, (d, d’) Golgi‐EGFP, (e, e’) Golgi‐mCherry, (f, f’) Lyn‐Venus, (g, g’) Lyn‐mCherry, (h, h’) EGFP‐Tuba, (i, i’) hEMTB‐EGFP, (j, j’) EB1‐EGFP, (k, k’) Venus‐Actin, (l, l’) Venus‐Moesin, (m, m’) EGFP‐Paxillin, and (n, n’) ZO1‐EGFP. (a‐n) images represent single Z‐sections from blastocyst shown in Figure 1, (a’‐n’) enlarged views of the areas boxed in (a‐n) without nuclear DAPI staining. Scale bar = 20 μm (a‐n), 5 μm (a’‐n’). [file DVG-57-na-s001.tif]

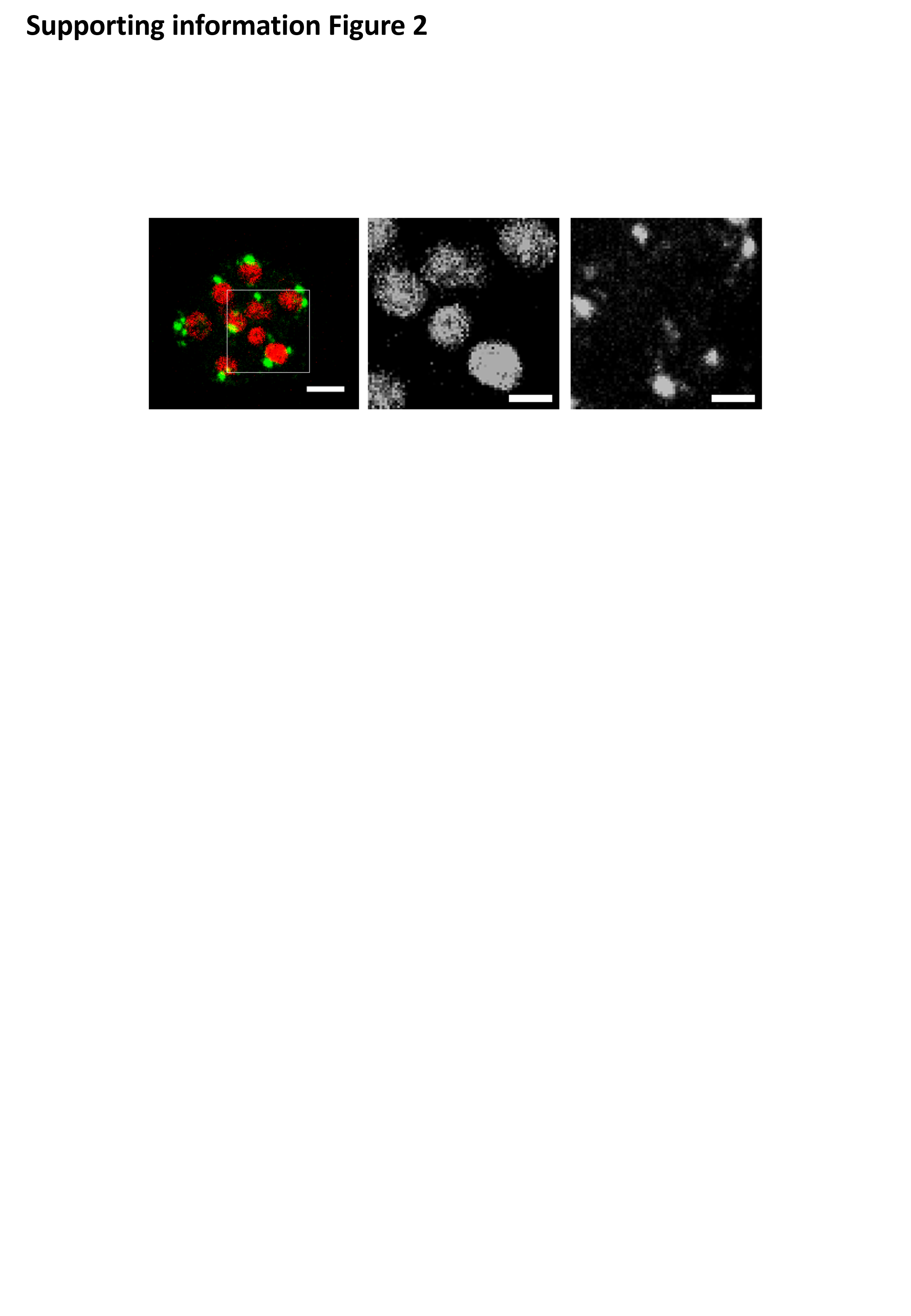

Supplement: Supplementary file 2 — Supporting information Figure 2 Expression of fluorescent fusion proteins in 8‐cell R26‐Golgi‐EGFP/H2B‐mCherry embryos. Golgi‐EGFP/H2B‐mCherry (left), H2B‐mCherry (middle) and Golgi‐EGFP (right). The left panel represents a single Z‐section from Figure 2, and the middle and right panels are enlarged views of the area boxed in the left panel. Scale bar = 20 μm (left), 5 μm (middle and right). [file DVG-57-na-s002.tif]

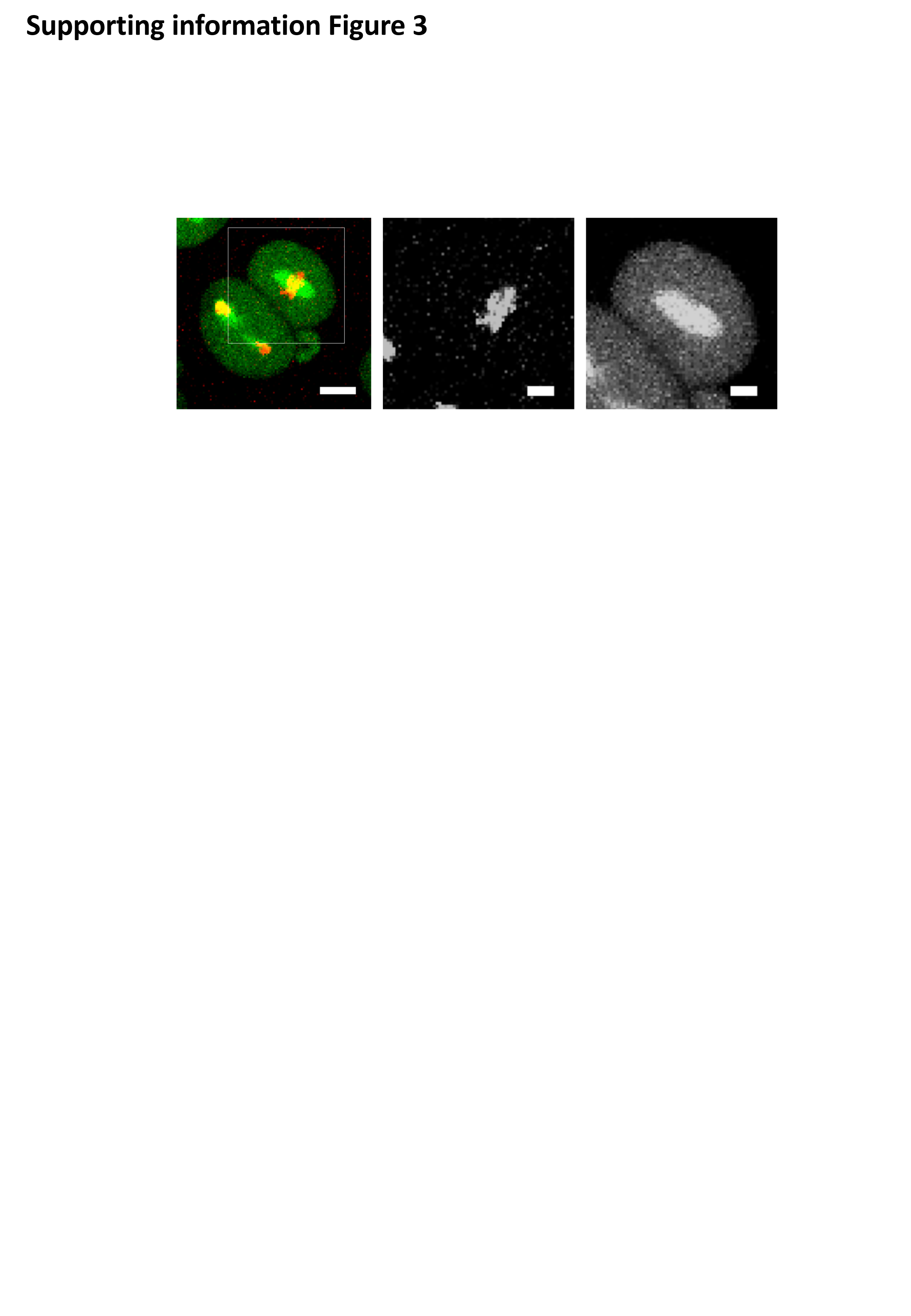

Supplement: Supplementary file 3 — Supporting information Figure 3 Expression of fluorescent fusion protein in 2‐cell R26‐EGFP‐Tuba/H2B‐mCherry embryos. EGFP‐Tuba/H2B‐mCherry (left), H2B‐mCherry (middle) and EGFP‐Tuba (right). The left panel represents a single Z‐section from Figure 3. The middle and left panels are enlarged views of the area boxed in the left panel. Scale bar = 20 μm (left), 10 μm (middle and right). [file DVG-57-na-s003.tif]

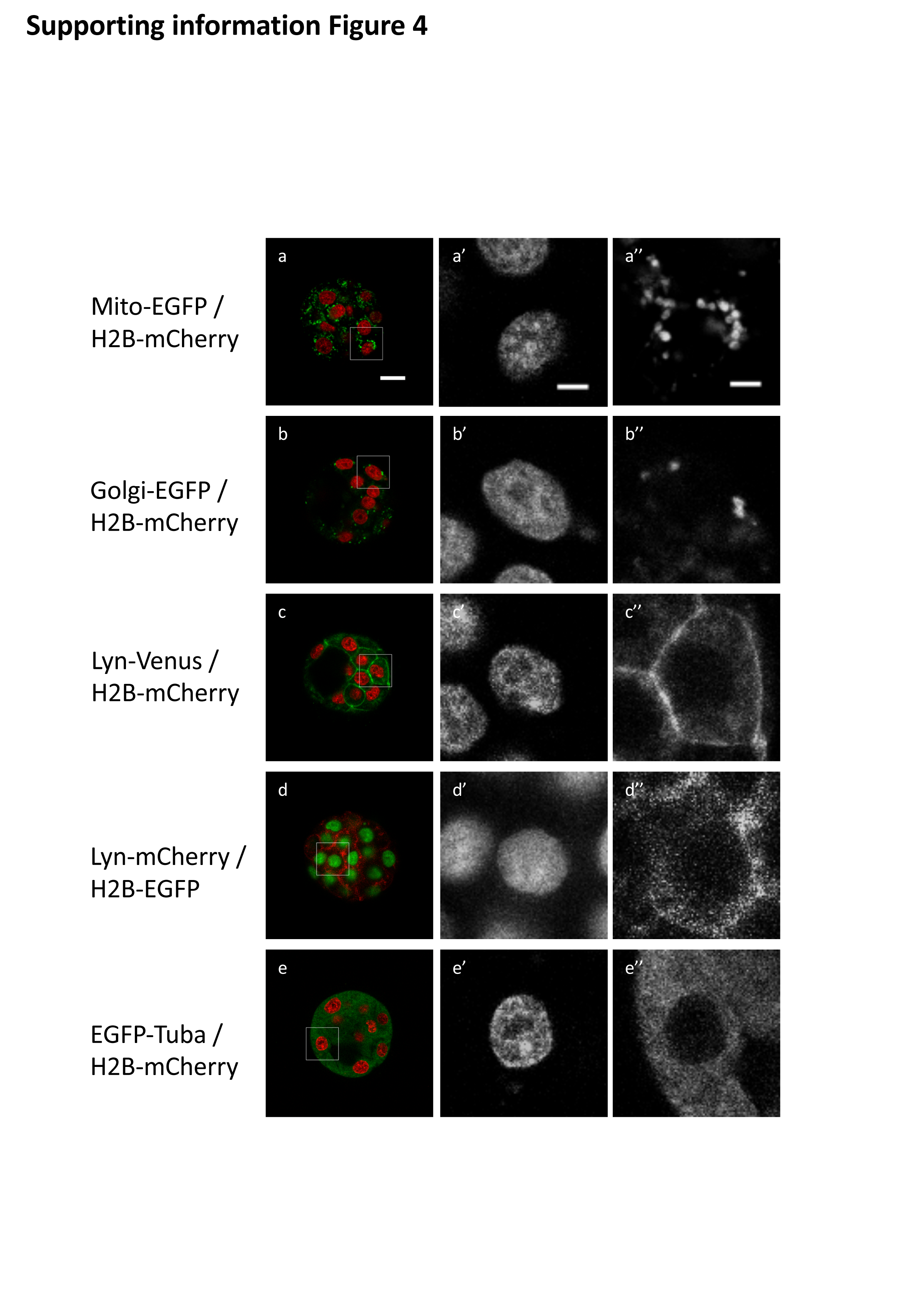

Supplement: Supplementary file 4 — Supporting information Figure 4 Expression of fluorescent fusion proteins in dual‐labeled blastocysts. (a) Mito‐EGFP/H2B‐mCherry, (a’) H2B‐mCherry, (a”) Mito‐EGFP, (b) Golgi‐EGFP/H2B‐mCherry, (b’) H2B‐mCherry, (b”) Golgi‐EGFP, (c) Lyn‐Venus/H2B‐mCherry, (c’) H2B‐mCherry, (c”) Lyn‐Venus, (d) Lyn‐mCherry/H2B‐EGFP, (d’) H2B‐EGFP, (d”) Lyn‐mCherry, (e) EGFP‐Tuba/H2B‐mCherry, (e’) H2B‐mCherry, (e”) EGFP‐Tuba. (a‐e) the images represent single Z‐sections from the blastocysts in Figure 4. (a’‐e’, a”‐e”) enlarged views of the areas boxed in (a‐e). Scale bar = 20 μm (a‐e), 10 μm (a’‐e’, a”‐e”). [file DVG-57-na-s004.tif]

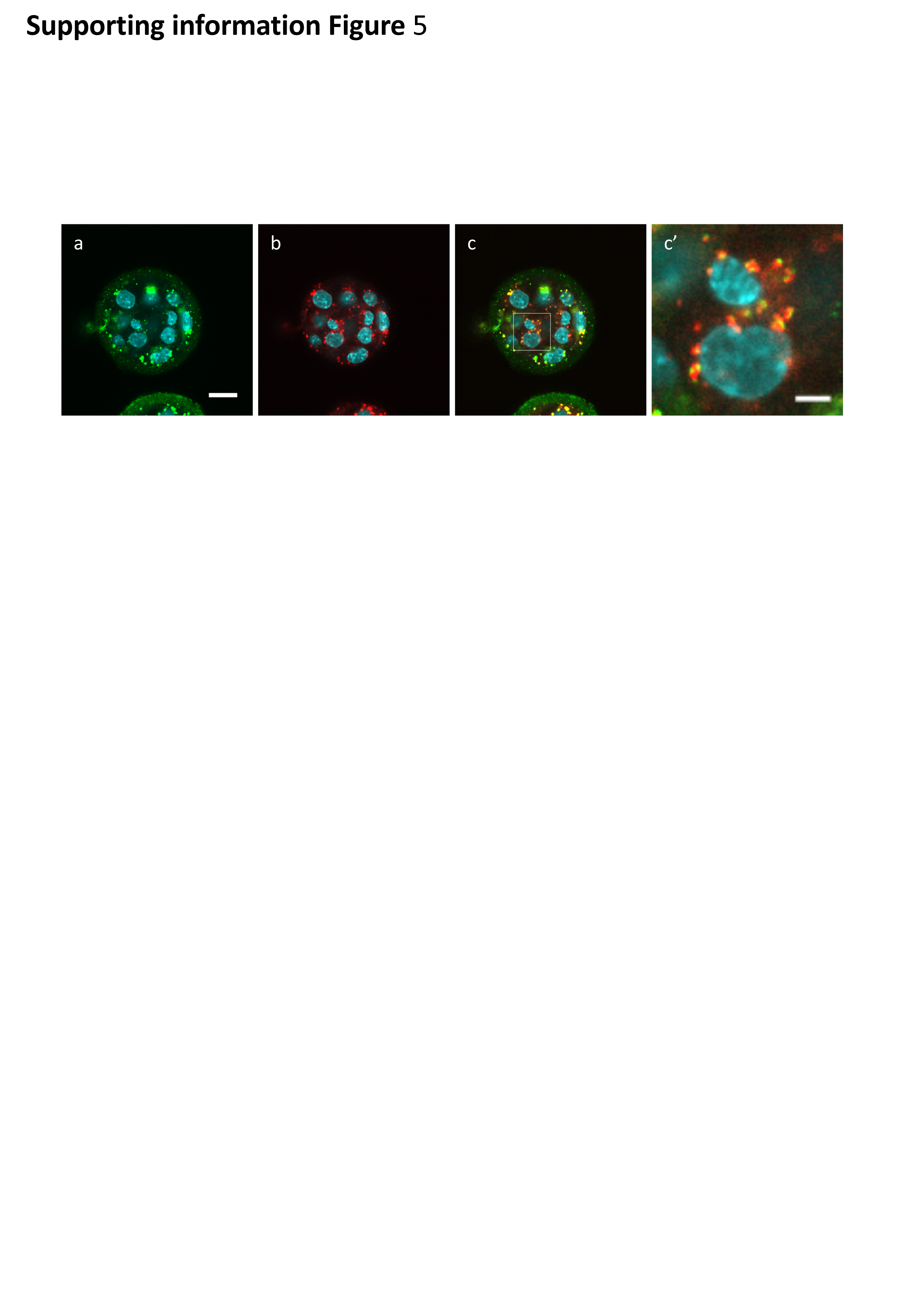

Supplement: Supplementary file 5 — Supporting information Figure 5 Immunostaining of Golgi apparatus in a Golgi‐mCherry blastocysts. (a) Immunostaining for Golgi apparatus. (b) Golgi‐mCherry fluorescent fusion protein. (c) merged image. (c’) an enlarged view of the area boxed in (c). Scale bar = 20 μm (a‐c), 5 μm (c’). [file DVG-57-na-s005.tif]

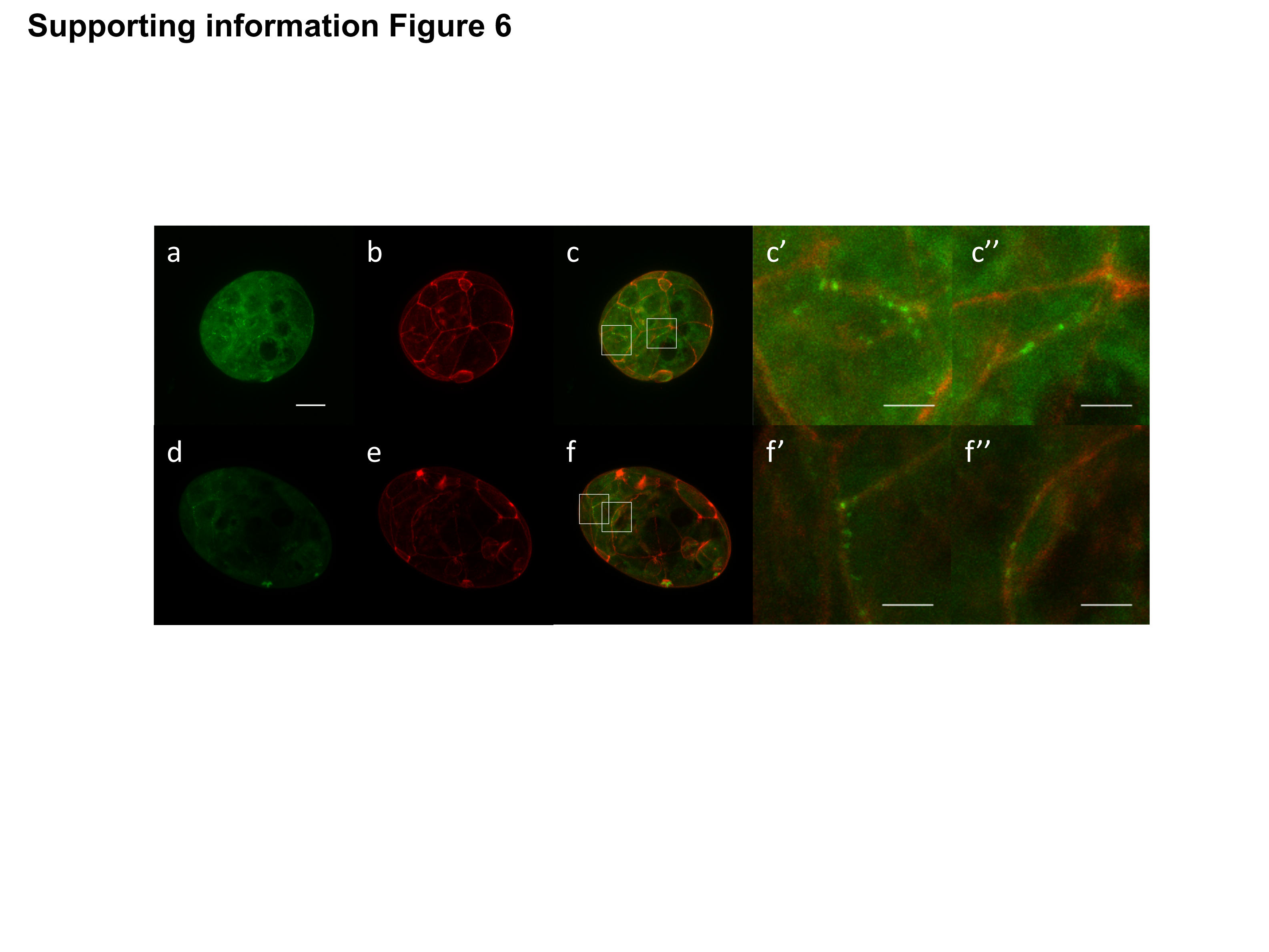

Supplement: Supplementary file 6 — Supporting information Figure 6 Expression of the fluorescent fusion protein in the blastocysts expressing R26‐EGFP‐Paxillin. (a, d) EGFP‐Paxillin. (b, e) phalloidin staining. (c, f) merged image. (c’, c”) enlarged views of the area boxed in (c). (f’, f”) enlarged views of the area boxed in (f). Images in (a–c) and (d–f) are MIPs. Scale bar = 20 μm (a–c and d–f), 5 μm (c’, c”, f’, and f”). [file DVG-57-na-s006.tif]

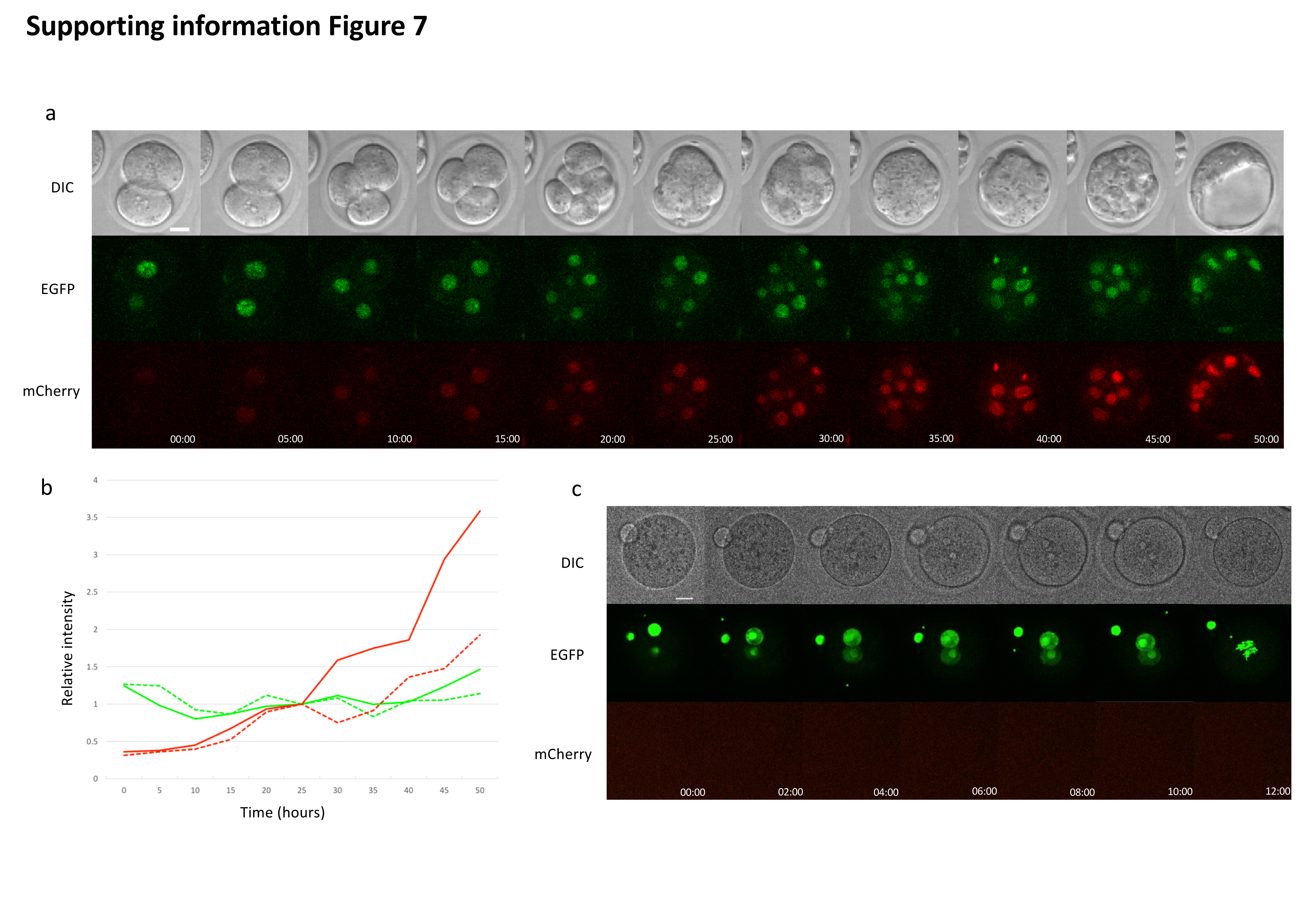

Supplement: Supplementary file 7 — Supporting information Figure 7 Changes of signal intensities in nuclei during pre‐implantation development. (a) Time‐lapse images of an embryo obtained by crossing female R26‐H2B‐EGFP and male R26‐H2B‐mCherry mice developing from the 2‐cell through blastocyst stages. Time‐lapse images were taken every 1 hour; selected images at 5‐hour intervals (i.e., every five images) are shown. The z‐step size is 5 μm. Scale bar = 20 μm. (b) Analysis of signal intensities of H2B‐mCherry and H2B‐EGFP from time‐lapse images in (a). Green solid and dotted lines indicate EGFP signal intensities of different blastomeres in the same embryo. Red solid and dotted lines indicate mCherry signal intensities of different blastomeres in the same embryo. The intensity of each signal at 25 hours was defined as 1.0, and each time point is expressed as a relative value. (c) Time‐lapse images of a developing embryo obtained by crossing R26‐H2B‐mCherry and R26‐H2B‐EGFP during the pronuclear stage. Embryos were obtained from the oviducts. Time‐lapse images were taken every 10 min; selected images at 2‐hour intervals (i.e., every 12 images) are shown. Time stamps are shown on the bottom. H2B‐mCherry and H2B‐EGFP images are MIPs. The z‐step size is 5 μm. Scale bar = 20 μm. [file DVG-57-na-s007.tif]
